# Supplementary material for: Patterns of Intron Gain and Loss in Fungi
Source: PLoS Biol. 2004 Nov 30;2(12):e422. doi: 10.1371/journal.pbio.0020422 (PMC532390; doi:10.1371/journal.pbio.0020422)
Supplement: Table S1 — Also available at http://genes.mit.edu/NielsenEtAl/. (4.3 MB ZIP). [file pbio.0020422.st001.zip › NielsenEtAl/html/553.html]

AN8763.1.NCU03156.1.MG02669.1.FG08566.1


```
 CLUSTAL W (1.82) Multiple Sequence Alignments - Introns Inserted


Sequence 1: MG02669.1	108 aa
Sequence 2: FG08566.1	94 aa
Sequence 3: AN8763.1	94 aa
Sequence 4: NCU03156.1	94 aa
Alignment Length: 108 aa
Number Identitical Residues: 46 aa
Alignment Score (without introns) 2350


MG02669.1 	MSAKYAFTQSLKEVRFLFCQTADHSAGVR2SFLMRAYPTMKKNNPTTPILIREAAGTIPK
NCU03156.1	MSGRFAFNKGLKEVRFLFCQTGEHSAATR2SFLLRNYPAMKKDNPATPILIRDASGTLPK
FG08566.1 	MSAKYAFTKSLREVRFLFCQTSEQSAALR2SFITRSYPTMKRNNPNIPILIREAAGTQPK
AN8763.1  	MSSKYVFTKGLKELRFLLCQTSEQSAATR2LFINRAYPTMKKHNPHTPILIREAAGTLPR
          	**.::.*.:.*:*:***:***.::**. *  *: * **:**:.**  *****:*:** *:

MG02669.1 	VYARYD~LGVEKSQSLEG1LSDKQIEDAVTGLVKQGH2IPKLQALVLLGGKS
NCU03156.1	VYARFE1FGKEKSQSLEG1LSDQQIEETVSSLVKNNS~--------------
FG08566.1 	IFARYE1RGVEKSQILEG~LSDKEIEDTVTSLVQPAQ~--------------
AN8763.1  	VYARYA1LGKEKVESLNG1LSDQQIEEKVKQLVKESS~--------------
          	::**:   * ** : *:* ***::**: *. **:
```
